# Prevalence of *Protoparvovirus carnivoran*1/Feline Coronavirus and Associated Risk Factors in Cats Admitted to a Public Shelter in Southern Italy

**DOI:** 10.3390/vetsci13060528

**Published:** 2026-05-29

**Authors:** Francesco Mira, Giulia Donato, Giorgia Schirò, Luigi Arcuri, Giuseppa Purpari, Elisabetta Giudice, Valentina Giannitrapani, Francesca Gucciardi, Antonina Princiotta, Annalisa Guercio

**Affiliations:** 1 Department of Agricultural, Food and Forest Science, University of Palermo, 90128 Palermo, Italy; francesco.mira@unipa.it; 2 Department of Veterinary Sciences, University of Messina, Polo Universitario dell’Annunziata, 98168 Messina, Italy; giudonato@unime.it (G.D.); elisabetta.giudice@unime.it (E.G.); 3 Istituto Zooprofilattico Sperimentale della Sicilia “A. Mirri”, 90129 Palermo, Italy; giuseppa.purpari@izssicilia.it (G.P.); francesca.gucciardi@izssicilia.it (F.G.); antonina.princiotta@izssicilia.it (A.P.); annalisa.guercio@izssicilia.it (A.G.); 4 Azienda Sanitaria Provinciale di Palermo, 90129 Palermo, Italyvalentina.giannitrapani@asppalermo.org (V.G.)

**Keywords:** *Protoparvovirus carnivoran*1, feline panleukopenia virus, canine parvovirus type 2, shelter medicine, cats, stray cats, colony cats, risk factors

## Abstract

This study aimed to evaluate the introduction of *Protoparvovirus carnivoran*1 (feline panleukopenia virus, FPV/canine parvovirus type 2, CPV-2) and feline coronavirus (FCoV) at admission in a public feline shelter, their molecular epidemiology, and potential associated risk factors. For this purpose, rectal swabs collected from a total number of 368 stray and colony cats were analyzed by a set of classical PCR assays for the detection, typing, and sequencing of FPV/CPV-2 (368 cats) and FCoV (198 cats). A statistical analysis (Chi-squared test or Fisher’s exact test) was performed to evaluate the association between FPV/FCoV positive results and associated data. FPV, CPV-2c, and FCoV-I were detected in 25.8%, 0.5%, and 30.8% of the tested cats, respectively. High degrees of nucleotide identity were observed with local, national, and international viral strains previously collected from cats, wild carnivores, and dogs. Presence of FPV was significantly more likely in male cats (*p* = 0.024; OR = 0.580) and in 6–12-month (*p* = 0.003; OR = 4.69) or 12–36-month (*p* = 0.004; OR = 2.4) cats, while presence of FCoV was higher in younger cats (<3 months) and in those showing respiratory signs (*p* = 0.046; OR = 2.76). Monitoring both viruses and their associated infection risk factors can help reduce the risks of introduction and transmission within feline colonies, veterinary clinics, or hospitals, supporting targeted measures and standards of care.

## Simple Summary

This study investigated the presence and introduction of parvovirus and feline coronavirus infections in cats admitted to a public animal shelter. The main problem addressed was the risk that infected stray and colony cats could introduce and spread these viruses within shelter environments. The aim was to determine how common these viruses were at admission, to study their genetic characteristics, and to identify factors that might increase the risk of infection. Rectal samples from 368 cats were tested using molecular methods to detect and characterize the viruses, and statistical analyses were used to explore associations with different variables. The results showed that the parvoviruses were present in 26.4% of the tested cats, while feline coronavirus was detected in 30.8%. Genetic analysis revealed close similarity with strains previously found in domestic and wild animals. Male cats and young adults were more likely to carry parvovirus, while very young cats and those showing respiratory illness were more frequently infected with feline coronavirus. The study concludes that systematic screening and monitoring of incoming shelter cats, together with attention to identified risk factors, can help prevent outbreaks and support safer management practices in shelters and veterinary settings.

## 1. Introduction

The domestic cat (*Felis silvestris catus*) is among the most widely kept and globally distributed companion animals. The exact size of the global feline population, as well as its distribution trends and incidence across different countries, has not been definitively established; however, it is reasonable to assume that these figures are substantial. Moreover, the characteristics of feline populations can vary considerably between countries, reflecting context-specific demographic and management differences. For example, it is estimated that in 2024 the number of owned cats in Italy could have reached almost 12 million, with an increase of over 1 million compared to previous estimates [1], making Italy one of the most “pet-friendly” countries. In addition, there is a large and unestimated population of stray cats (feral cats and feral cat colonies) integrated into urban areas, and small niches hosting European wildcats (*Felis silvestris silvestris*), such as in Sicily, southern Italy [2], overall contributing to the definition of the national feline population.

As a result of this increasingly close relationship between society and cats, over the years, even greater efforts have been made to manage stray and free-roaming pets in this country, and an increasingly broad national and regional regulatory framework has established laws for their protection. In accordance with this framework, regional public health shelters are established for the care and sterilization of stray animals and cats from managed colonies [3,4]. Ensuring cats can live freely in their natural habitats requires facilitating both their movement through public shelters and their subsequent return, while also protecting the health of their native environments and cohabiting animal species. To pursue these objectives of the management and protection of cats in urban environments, health monitoring and prevention of the onset of species-specific diseases play a functional role. This monitoring also demonstrates its value and usefulness in relation to the close relationship between the cat’s free-roaming lifestyle and the barrier-free environment in which it lives, which can, therefore, favor the conditions for the direct or indirect spread of pathogens through shared environments between susceptible species.

In this context, cats are usually admitted to shelters to ensure adequate care or for sterilization, and while their health status is assessed upon admission, the health and safety of both them and other animals in the shelter must be maintained during their stay in shelters [5]. Moreover, as in many European countries, the Italian legislature has adopted so-called ‘no-kill’ policies, limiting the euthanasia of animals introduced into shelters only for specific, documented, and limited health and behavioral reasons: reducing the number of unnecessary animal deaths and implementing interventions that increase cat adoptions or their reintroduction into their environments of origin is therefore desirable in order to comply with this framework [6,7]. The causes of infection or mortality in dog and cat shelters have been investigated, mostly limited to observational studies during shelter stays and on dogs, but specific data on cats are still limited [7].

Preventing pathogens from entering the shelter environment is challenging and commonly relies on the implementation of appropriate quarantine, isolation, hygiene, and veterinary care procedures. Prevention is particularly necessary for the most common and severe feline viral diseases, such as feline panleukopenia (FPV) and feline coronavirus (FCoV), due to the impact they can have in these environments.

Feline panleukopenia is caused by the *Protoparvovirus carnivoran*1 (family *Parvoviridae*; subfamily *Parvovirinae*; genus *Protoparvovirus*) (https://ictv.global/taxonomy, accessed on 13 February 2026), whose viral species include the feline panleukopenia virus and canine parvovirus type 2 (CPV-2), affecting both domestic and wild cats, with FPV being the most commonly detected virus in affected animals [8]. *Protoparvovirus carnivoran*1 is a small, non-enveloped, single-stranded DNA virus, transmitted via the fecal-oral route through direct contact with infected animals or indirectly via inanimate contaminated objects (fomites); it is characterized by resistance and long persistence in the environment (from weeks/months up to a year). Both close contact with infected animals or fomites, particularly in overcrowded environments, and its long resistance in the environment are conditions contributing to its spread [8,9]. The viral genome (about 5200 bases in length) encodes for two non-structural (NS1 and NS2) proteins and for two structural (VP1 and VP2) proteins: VP2 is the major capsid component and serves as the main determinant of the virus’s antigenicity, pathogenicity, and host range [10]. FPV and CPV-2 are more than 98% identical at the DNA sequence level; FPV differs from the original CPV-2 type and its variants (CPV-2a, CPV-2b, and CPV-2c) by only a few amino acid substitutions in the VP2 viral protein, some of which are responsible for relevant biological and antigenic changes (K80R, K93N, V103A, D323N, N564S, A568G; M87L, I101T, A300G, D305A, N426D/E) [11,12].

Feline coronavirus (family *Coronaviridae*; subfamily *Orthocoronavirinae*; genus *Alphacoronavirus*; subgenus *Tegacovirus*; species *Alphacoronavirus suis*) (https://ictv.global/taxonomy, accessed on 13 February 2026) is a large, enveloped, single-stranded RNA virus. Starting from the 5′-end, two-thirds of the coronavirus genome consists of two overlapping open reading frames (ORF1a and 1b), while the other third consists of ORFs encoding structural proteins: the spike (S), membrane (M), nucleocapsid, envelope, and the non-structural accessory (3a, 3b, 3c, 7a and 7b) proteins [13]. Based on genomic properties and antigenicity, FCoV is divided into types I and II [14], and both types can exist as two different pathotypes (biotypes), referred to as feline enteric coronavirus (FECV) (which mainly replicates in the enteric epithelial cells and does not often cause clinical signs or, occasionally, causes enteritis) and feline infectious peritonitis virus (FIPV) (which replicates efficiently in systemic monocytes or macrophages, and results in a lethal infection) [13,15]. Mutations in different genes have been postulated to be associated with the evolution from the less-virulent intestinal FCoV into the virulent systemic virus associated with FIP, among which two alternative amino-acid differences (M1058L and S1060A) lie in the S2 fusion domain of the S protein and were likely associated with the development of FIP [13,16]. Playing a significant role in the classification, evolution, host adaptation, and viral functions, both the S and M genes are usually used as targets for molecular epidemiological surveillance [17]. FCoV infection is transmitted via the fecal-oral route, and infected cats shed the virus for days, weeks/months, and even, in some cases, for their entire lives. Unfortunately, a small proportion of FCoV-infected cats develop FIP, and certain conditions (stress, shelter housing, overcrowding, surgery, or neutering) may contribute to the development of FIP in FCoV-infected cats [13].

This study was conducted on domestic cats at the time of admission for neutering or primary care in a public feline shelter with a high turnover of stray and colony cats, with the aim of evaluating the introduction of *Protoparvovirus carnivoran*1 and feline coronavirus at admission, their molecular epidemiology, and potential risk factors associated with their introduction into shelters. These data may be helpful when considering resource allocation to minimize the potential harm to shelters and cat colonies due to the spread of highly resistant viruses that cause severe, potentially fatal diseases.

## 2. Materials and Methods

### 2.1. Study Design, Clinical Examination and Sampling

Rectal swabs were conducted for the study: they were routinely collected between 2 March 2023 and 31 December 2024 from cats being admitted to a leading regional municipal shelter in Palermo (Sicily region, southern Italy). Cats were enrolled independently of breed, sex, age, neutering status, or clinical presentation to ensure a representative study population. Exclusion criteria included unavailable or inadequate samples and incomplete epidemiological or clinical records, which prevented statistical analysis. Sampled cats were strays or lived in registered colonies within the Palermo municipality that were introduced into the public shelter to ensure adequate care or for neutering/spaying. Each cat underwent a clinical examination. Data on reason for hospitalization, origin, age group, gender/neutering status, and clinical signs were recorded in a clinical form, following a categorization synthesized in Table 1. Rectal swabs, along with associated data, were then submitted to the laboratory within 24 hours of collection for analysis and tested to detect the presence of FPV/CPV-2 and FCoV. The introduction and subsequent sampling of cats were halted between 7 June and 30 June 2024, while FCoV testing was suspended from 7 July 2023 to 18 March 2024 and from 9 to 31 August 2024 for organizational reasons. For these reasons, a total of 368 and 198 cats were then tested for FPV/CPV-2 and FCoV, respectively.

### 2.2. Sample Processing and DNA/RNA Extraction

A total volume of 2 mL of a culture medium (Eagle’s Minimum Essential Medium) supplemented with an antibiotic and antimycotic solution was added to each swab. Swabs were then maintained at 37 °C for 60 minutes; suspensions were then transferred into a 15 mL sterile tube and centrifuged at 1500× *g* for 15 min at 4 °C. Supernatants were finally stored at −80 °C.

DNA and RNA were extracted respectively from 200 μL and 140 μL of supernatants, using the DNeasy Blood & Tissue Kit (Qiagen S.p.A., Hilden, Germany) and QIAmp Viral RNA Mini Kit (Qiagen S.p.A.), respectively, according to the manufacturer’s instructions. Extracted DNA and RNA were stored at −80 °C until processed.

### 2.3. FPV/CPV-2 Detection and Typing

Extracted DNA was tested by a traditional PCR assay for the detection of *Protoparvovirus carnivoran*1 species, including both feline panleukopenia virus (FPV) and canine parvovirus type 2 (CPV-2), using a primer pair described by Touihri and colleagues [18], targeting the VP2 gene sequence (700 nucleotides in length). FPV/CPV-2 detection was carried out with a GoTaq^®^ G2 DNA Polymerase Kit (Promega Corporation, Madison, WI, USA) in a 50 μL reaction mix (Supplementary Material—Table S1). A field CPV-2c strain (code: VIR AR 12511 1), obtained from the Biobanca del Mediterraneo, Italy (www.bbmed.it, accessed on 6 February 2026), was used as a positive control.

Viral typing of parvoviruses was based on the analysis of the VP2 gene sequence and of the amino-acid (aa) residues discriminating the viral type (FPV or CPV-2) and the CPV-2 variants [19].

### 2.4. FCoV Detection and Typing

For FCoV detection, extracted RNA was tested by a traditional RT-PCR assay, using a primer pair described by Campolo and colleagues [20], targeting the matrix gene sequence (409 nucleotides in length). FCoV detection was carried out with a QIAGEN^®^ OneStep RT-PCR Kit (Qiagen S.p.A.) in a 50 μL reaction mix (Supplementary Material—Table S1). A canine coronavirus RNA (canine coronavirus, ATCC 809-VR) was used as a positive control.

For FCoV genotype prediction, RNAs which tested positive to the above-described detection protocol were further tested by a reverse transcription nested PCR (RT-nPCR) assay, using two primer sets targeting the spike (S) gene, described by Addie and colleagues [21], in two separate 25 μL reaction mixes (Supplementary Material—Table S2). A canine coronavirus RNA (canine coronavirus, ATCC 809-VR) was used as a positive control.

To amplify a fragment of the Spike gene sequence, including codons for residues of the S protein affected by mutations M1058L and S1060A associated with the FCoV pathotype change (FECV/FIPV), FCoV-positive RNAs were further tested by a one-step RT-PCR assay using a SuperScript™ One-Step RT-PCR System with Platinum™ Taq DNA Polymerase kit (ThermoFisher Scientific) and two primer pairs described by Decaro and colleagues [22], in 25 μL reaction mixes (Supplementary Material—Table S2).

### 2.5. Sequence and Phylogenetic Analyses

Amplicons of the expected size (obtained via FPV/CPV-2 detection and from FCoV detection and typing assays) were purified with an Illustra™ GFX™ PCR DNA and Gel Band Purification Kit (GE Healthcare Life Sciences, Amersham, Buckinghamshire, UK) and submitted to BMR Genomics srl (Padova, Italy) for both-strand Sanger sequencing. Sequences were assembled and analyzed using BioEdit ver 7.0.5.3 software [23]. Obtained sequences were compared with homologous sequences using online databases by BLASTn (https://blast.ncbi.nlm.nih.gov/Blast.cgi, accessed on 5 and 11 November 2025) [24]. After removing identical sequences, a total of 132 sequences were submitted to the DDBJ/EMBL/GenBank databases with accession numbers PZ012441–PZ012460 (*Protoparvovirus carnivoran*1) and PZ012461–PZ012572 (feline coronavirus) (listed in Supplementary Material—Table S3).

Phylogenetic analysis was carried out with related sequences obtained from the GenBank database using MEGA X ver. 10.2.6 software [25] to build a Maximum Likelihood (ML) phylogenetic tree selecting the substitution model with the lowest Bayesian Information Criterion (BIC) (CPV-2/FPV: Tamura 3-parameter model + gamma distribution + invariable sites, T92+G+I; FCoV detection: Tamura 3-parameter model + gamma distribution + invariable sites, T92+G+I; FCoV genotyping: Tamura–Nei-parameter model + gamma distribution, TN93+G; partial FCoV S gene sequencing: General Time Reversible model + gamma distribution + invariable sites, GTR+G+I) and bootstrap analyses with 1000 replicates.

### 2.6. Statistical Analysis

Statistical analysis was performed using Jamovi 2.3.28.0 statistical software. The distribution of continuous variables was evaluated by the Shapiro–Wilk test and descriptive statistics were obtained for all the investigated variables. Chi-squared test or Fisher’s exact test were used to evaluate the association between FPV/CPV-2, FCoV positivity or their coinfection and reason for hospitalization (neutering, trauma, pathology), origin (stray or colony cats), gender/neutering status (intact male, intact female, spayed male, neutered female), age (less than 3 months, 3–6 months, 6–12 months, 12–36 months, more than 36 months) and clinical signs (healthy cats, gastrointestinal, respiratory signs, trauma, or miscellaneous). Differences were considered significant if *p* values were <0.05.

## 3. Results

### 3.1. Cat Demographic and Clinical Data

Samples and data from a total of 368 cats were examined. A total of 208 stray cats and 160 cats from colonies were included in the study, involving 172 male (n = 146 intact males, n = 26 spayed males) and 196 female (n = 174 intact females, n = 22 neutered females) cats. Most of the cats belonged to the 3–6-month (n = 117) and 12–36-month (n = 151) age groups, while fewer cats belonged to the > 36-month (n = 69), 6–12-month (n = 21), and <3-month (n = 10) age groups. The most frequent reason for hospitalization was the occurrence of a pathology (71.2%), followed by trauma (20.9%) and neutering (7.9%). Based on clinical examination, 36 cats were clinically healthy, while 332 cats exhibited various clinical signs. These were categorized as follows: gastrointestinal (n = 67), respiratory (n = 134), respiratory and gastrointestinal (n = 17), trauma (n = 77), and miscellaneous (n = 37).

**Figure 1:**
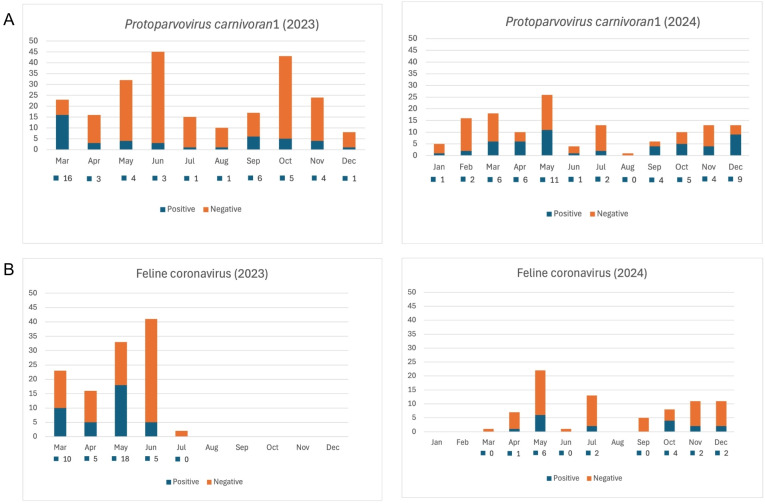
Monthly distribution of positive and negative PCR/RT-PCR screening results for *Protoparvovirus carnivoran*1 (**A**) and feline coronavirus (**B**) during 2023–2024.

A total of 368 and 198 cats were tested for FPV/CPV-2 and FCoV, respectively. A total of 26.4% (97/368) and 30.8% (61/198) of cats tested positive for FPV/CPV-2 and FCoV, respectively. FPV/FCoV coinfection, evaluated in a total of 198 cats, was detected in 21 cats. The positive *Protoparvovirus carnivoran*1 and feline coronavirus results obtained during the considered timeframe are depicted in Figure 1.

### 3.2. Protoparvovirus carnivoran1 Typing and Sequence Analysis

All but six amplicons obtained from the *Protoparvovirus carnivoran*1 screening were successfully sequenced, obtaining partial VP2 gene sequences of a total length of 700 nucleotides (including VP2 amino acid residues 284–515). According to the analysis of VP2 gene sequence and to the aa residues, a total number of 91 sequences was typed, revealing the presence of the feline panleukopenia virus (n = 89; 97.8%) and of the CPV-2c variant (n = 2; 2.2%).

Feline panleukopenia virus sequences showed high degrees of reciprocal nucleotide identity (ranging between 99.8 and 99.0%): according to this low heterogeneity, sequences were clustered into 18 different nucleotide sequence types (ntSTs) (Supplementary Material—Table S4). Overall, ntSTs showed high degrees of nucleotide identity with the sequences of FPV strains previously detected in Italy: in cats in Sicily in 2015 (acc.nr. KX434461) [26] and 2021/2022 (PP351396, PP351401, PP351432) [2] (100–99.43%), as well as in a northern Italian region (Veneto) in 2011 (MW847155, MW847158), 2016 (MW847174), and 2017 (MW847180, MW847181, MW847187) [27] (99.86–99.29%); in a golden jackal (Canis aureus) in Friuli Venezia Giulia (northern Italy) in 2011 (OP588006) [28] (99.57–99.29%) (more details in Supplementary Material—Table S4). All FPV sequences showed the aa discriminating between this viral type and the CPV-2 variants (300A, 305D, 323D), while additional single aa changes (N292Y, S293F: in n = 1 sequence; Q309L: in n = 1 sequence; L506I: in n = 5 sequence) were observed in comparison with the FPV reference strain (acc.nr. KX943311) (Table 2).

Similarly, CPV-2c sequences showed a high degree of nucleotide identity (100%) with the sequences of CPV-2c strains already detected in Italy, both in dogs in Sicily in 2020/2022 (OR463584, OR463658, OR463670) [29] and in Veneto in 2021 (OP588002) [28] (Supplementary Material—Table S4). Both CPV-2c sequences showed I and R at 324 and 370 aa residues, respectively, common to the CPV-2c strains originated in Asian countries and currently circulating in other continents, mainly in domestic dogs but also in domestic and wild carnivorans (Table 2).

**Figure 2:**
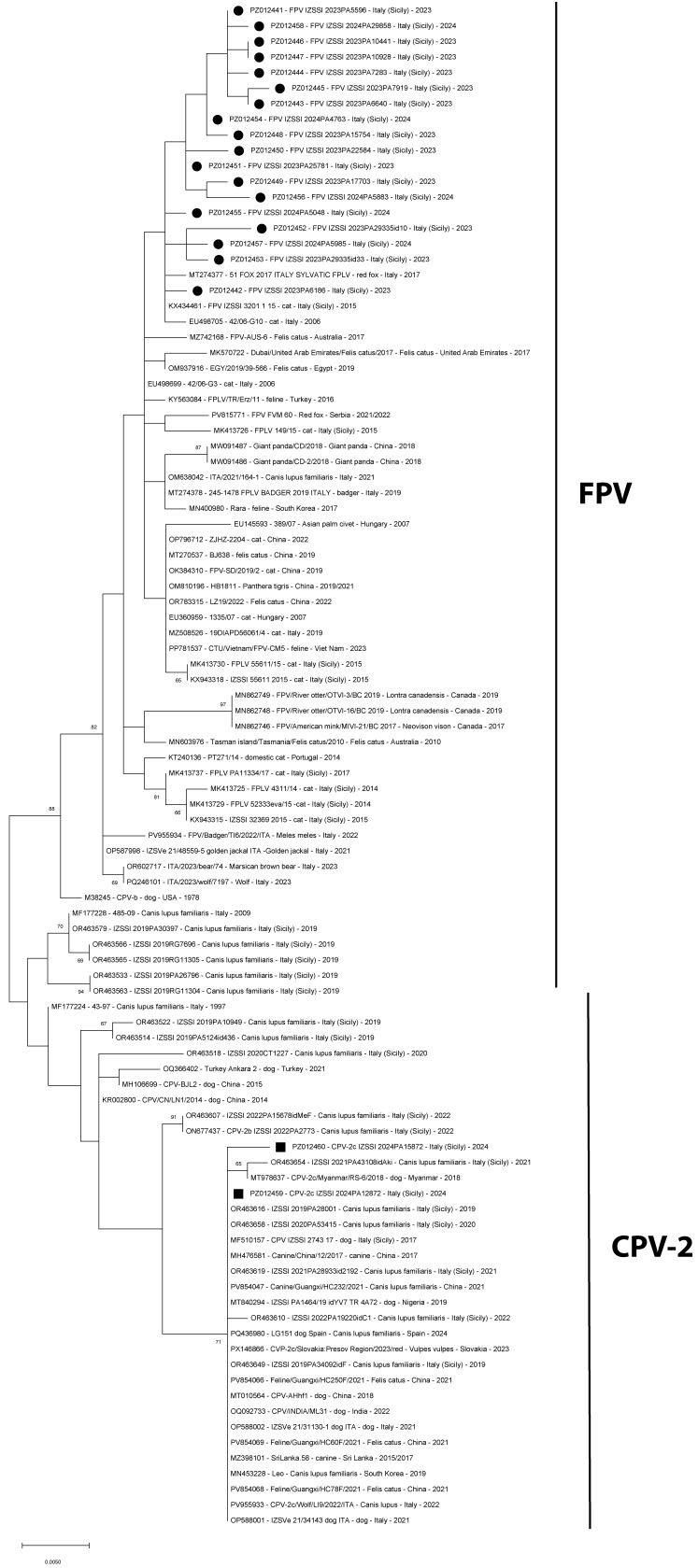
Maximum-likelihood tree (bootstrap values greater than 65 are shown) based on 98 partial VP2 gene sequences of feline panleukopenia virus and canine parvovirus type 2 strains. Black dots indicate FPV strains and black squares indicate CPV-2 strains, both analyzed in this study. Each sequence is indicated with accession number—strain/isolate name—animal species, country (region when Sicily) of origin—year of collection.

The topology of the phylogenetic trees based on the partial VP2 gene sequences showed that sequences of FPV and CPV-2c strains described in this study were included in separate branches, along with the sequences of other FPV and CPV-2 strains, respectively (Figure 2). Despite the overall low bootstrap values, as expected, FPV sequences clustered along with those of FPV strains detected in domestic and wild felines, as well as the CPV-2c sequences clustered with those of “Asian-like” strains detected in domestic dogs and cats and in other wild animals (Figure 2).

Overall, these sequence and phylogenetic data suggest a close connection between the detected strain and those circulating in the local feline and canine population, with a consequential introduction into the local municipal shelter through live cats, and a relationship with parvoviruses shared between the domestic/wild interface, suggesting a potentially direct or indirect bi-directional transmission.

### 3.3. Feline Coronavirus Typing and Sequence Analysis

**Figure 3:**
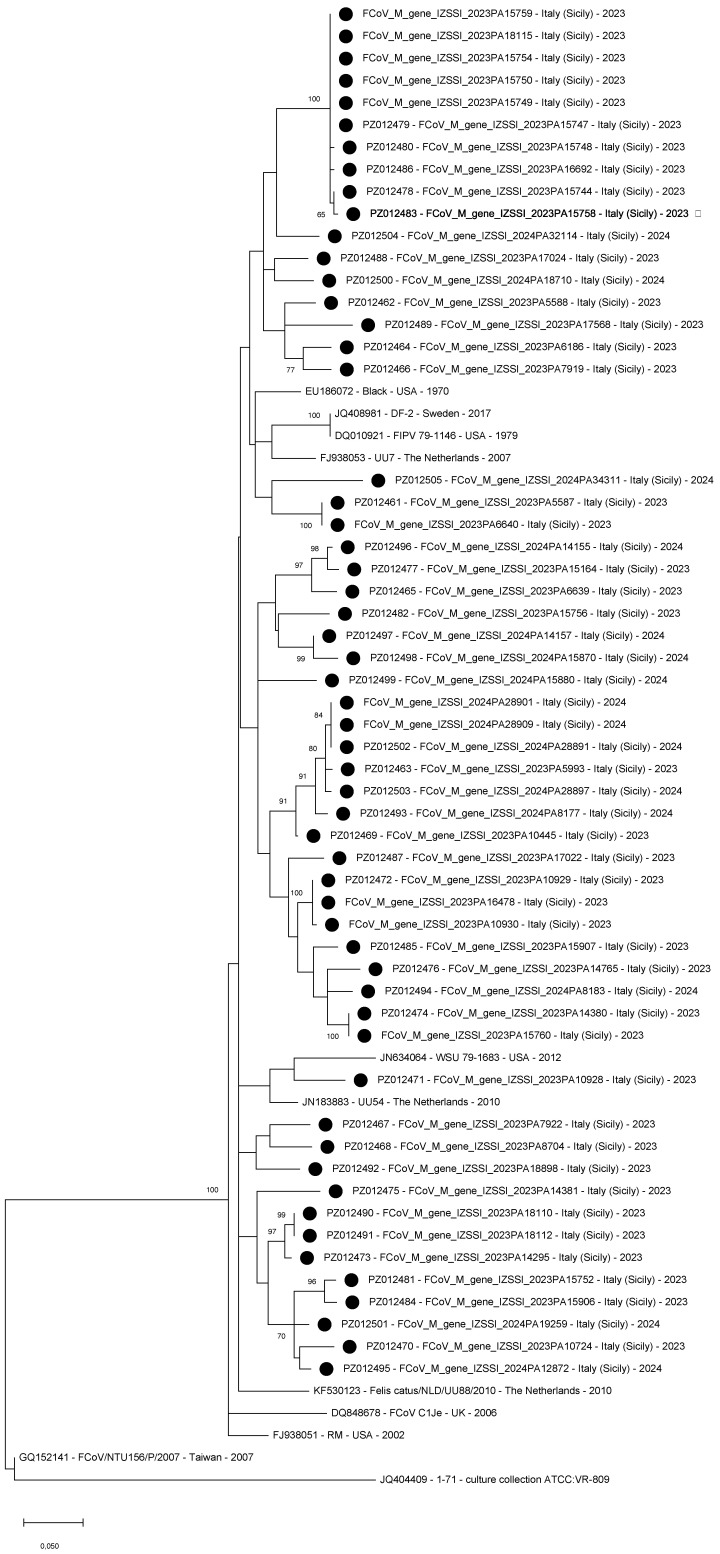
Maximum-likelihood tree (bootstrap values greater than 65 are shown) based on 67 partial M gene sequences of feline coronavirus. Black dots indicate FCoV strains analyzed in this study. Each sequence is indicated with accession number—strain/isolate name—country of origin—year of collection.

All but five amplicons obtained from the FCoV screening were successfully sequenced, obtaining n = 56 partial M gene sequences of a total length of 410 nucleotides. As expected, FCoV M gene sequences showed a certain degree of heterogeneity, showing reciprocal nucleotide identities ranging between 100 and 90.7%, and they clustered altogether with reference strains, without a statistically well-supported phylogeny (Figure 3).

**Figure 4:**
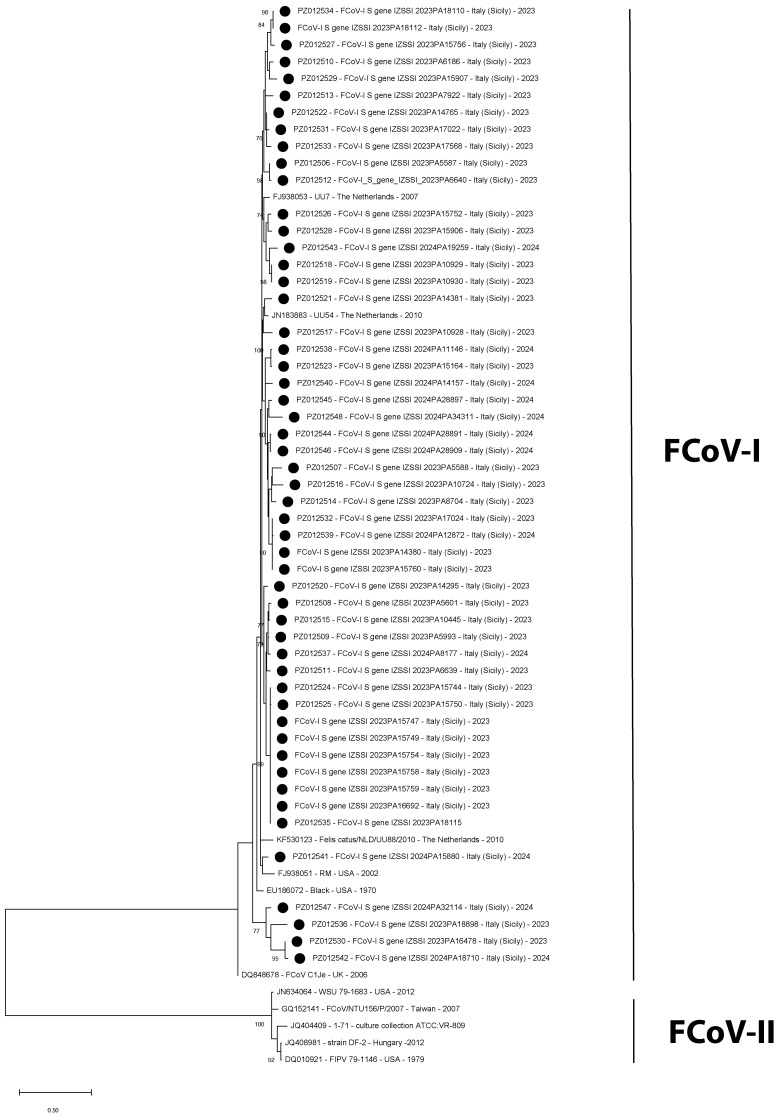
Maximum-likelihood tree (bootstrap values greater than 65 are shown) based on 63 partial S gene sequences of feline coronavirus. Black dots indicate FCoV strains analyzed in this study. Each sequence is indicated with accession number—strain/isolate name—country of origin—year of collection.

FCoV genotype prediction was determined for 52 out of 61 tested FCoV strains, and all were characterized as FCoV-I. Partial FCoV S gene sequences analyzed for the genotype prediction (360 nts) showed reciprocal nucleotide identities ranging between 100 and 81.6%. All FCoV sequences of this study, as well as the FCoV reference strain sequences, clustered in the phylogenetic tree in two separate branches according to the genotype (Figure 4).

Mutation analysis of the 1058 and 1060 aa residues of the S protein was carried out for 42 sequences: amino acid 1060S was observed in all sequences, while 1058M was observed in 41 sequences and 1058L in a single sequence (id. FCoV_S_gene_IZSSI_2023PA6186, acc.nr. PZ012553). Due to the low reading quality at the 5′-end, one sequence was excluded from the downstream analyses. Partial FCoV S gene sequences analyzed for mutation analysis (215 nts) showed reciprocal nucleotide identities ranging between 100 and 86.9%. The phylogenetic tree was built on a 189-nt fragment of the S protein to include the related sequences previously detected in Italy (highlighted in the figure with white dots) and, as expected, it showed a clustering according to the FCoV genotype, without a marked definition of sub-clades according to the origin or year of collection (Supplementary Material—Figure S1).

### 3.4. Association with Clinical Variables and Risk Factors

Risk factors and variables associated with FPV, FCoV positivity, and FPV/FCoV coinfection are reported in Table 3 and Table 4. The positivity for FPV was significantly associated with male cats (*p* = 0.024; OR = 0.580; 95% CI = 0.363–0.926) and in 6–12-month (*p* = 0.003; OR = 4.69; 95% CI = 1.75–12.6) or 12–36-month (*p* = 0.004; OR = 2.4; 95% CI = 1.32–4.38) cats compared to younger cats (3–6-month group). No association was found between FPV positivity and origin, reason for hospitalization, or clinical signs.

Positivity for FCoV was significantly associated with younger cats (<3 months) compared to 3–6 months (*p* < 0.001; OR = 23.4; 95% CI = 2.73–201), 6–12 months (*p* = 0.003; OR = 30; 95% CI = 2.63–343), 12–36 months (*p* < 0.001; OR = 20.6; 95% CI = 2.49–170) and > 36 months (*p* < 0.001; OR = 34.5; 95% CI = 3.63–328) age groups. Moreover, FCoV positivity was significantly associated with the presence of respiratory clinical signs (*p* = 0.046; OR = 2.76; 95% CI = 1.05–7.24). No association was found between FCoV positivity and origin, gender, or reason for hospitalization.

Coinfection was significantly associated with stray cats (*p* = 0.002; OR = 5.67; 95% CI = 1.61–19.9) and cats with respiratory clinical signs (*p* = 0.019; OR = 8.30; 95% CI = 1.04–66.1). No association was found between coinfection and gender, age, or reason for hospitalization.

## 4. Discussion

Both parvovirus and feline coronavirus infections are widespread among domestic cats worldwide. Although less reported, these infections are widespread either in stray cats in urban and suburban areas or in feral cats and other wildlife, some of which are recognized as endangered or threatened species [30,31,32,33]. Their persistence, both in domestic and wild animal populations, is conditioned by direct or indirect transmission via the fecal-oral route and by their intrinsic resistance in the environment, which ranges from days/weeks (for coronaviruses) to months (for parvoviruses) [9,34]. These are also key factors in the introduction, spread, and persistence of both viruses in feline shelters; in this environment, cofactors (such as overcrowding, stressors, comorbid conditions, poor biosecurity and management measures, and low protective immune coverage) can contribute to the development of clinical cases, ranging from mild to severe and sometimes with negative outcomes.

For this reason, it is of paramount importance to monitor both these viruses and the associated infection risk factors to reduce the risks of viral introduction and transmission and improve the health and well-being of shelter cats. Indeed, some authors have suggested that the number of dead and euthanized cats in shelters is highest in the initial period (within the first six months) after shelter admission [35], thus deserving targeted measures and standards of care in the management and prevention of infectious diseases [20,36,37]. Most previous studies have relied on collected or recorded data on FPV/FCoV detection and risk variables associated with infection, within variable time intervals consecutive to admission to the facility, both in public shelters and breeding farms, with limited studies carried out at the time of entry to determine any specifically related risk factors [38,39]. In this study, we specifically focused on the time of admission of stray cats or colonies to shelters, linking free-roaming lifestyle and likely environmental factors to the context of feline healthcare; at this stage, we can pursue epidemiological objectives and analyze associated risk factors to guide and improve prevention management.

While it may be considered economically relevant in some contexts, adequate biosecurity measures are essential in feline shelters. In fact, according to the standards of care for incoming cats, a complete health check by a veterinarian is recommended, as well as specific screening and diagnosis for the most common contagious diseases [36,37]. In some of these cases, molecular tests can provide a rapid laboratory diagnosis and may add some relevant epidemiological data. In this study, 26.4% and 30.8% of the cats tested positive for parvovirus or FCoV, respectively. The occurrence of positive results was observed during almost all periods considered (Figure 1). These results highlight the potential risk of virus introduction posed by cats admitted to the shelter, thereby identifying the facility as a valuable observatory for monitoring the circulation of these viruses in their original environments.

As expected, almost all parvoviruses detected were typed as feline panleukopenia virus, the most common parvovirus in the domestic feline host. Genomic analysis of these viral strains, based on the VP2 gene fragment, showed low nucleotide heterogeneity, with only a few single amino acid changes, and high nucleotide identity with previously detected FPV strains in the same region [2]. These findings suggest the central role of the environment and feral cat populations in maintaining and introducing this virus to shelters. Therefore, entry procedures are a critical issue in shelter management, underscoring the importance of adequate biosecurity measures such as quarantine and isolation areas.

Although low in incidence, two strains of CPV-2 were detected, and were typed as the CPV-2c variant. These viral strains have clustered with others within a lineage of Asian origin, now widespread in many countries, including Europe and other Western continents. The key factors that influenced the rapid spread of this lineage are not fully understood, although the long-distance transport of dogs may have contributed. Similarly to what has been observed in the last year in Asian countries, where the CPV-2c strain belonging to the Asian lineage has been detected in domestic cats (Thailand [40], China [41,42], and India [43]) or in free-living leopard cats (Taiwan [44])—and these strains have been linked to those circulating in domestic dogs—a similar pattern of probable direct or indirect transmission between species (dog–cat and domestic–wild carnivores) has also been observed in Italy and Spain (Europe) [2,45], and has now been linked to the introduction into a feline shelter with free-living domestic cats.

Positive FPV test results were significantly associated with male cats and cats aged 6–12 months or 12–36 months compared to younger cats (3–6-month group). No associations were found between FPV positivity and other variables considered (origin, reason for hospitalization, or clinical signs). The most surprising finding was the association with cats aged 6 to 36 months, as infection in domestic cats is commonly expected in younger cats, linked to the decline of maternally derived antibodies and the lack of vaccine-induced immunity, making them highly susceptible. The statistical association of positive FPV test results in older cats suggests probably different epidemiological dynamics in free-living cats compared to domestic ones, which deserve clarification to possibly promote an age-specific approach and measures.

Positive results for FCoV showed a slightly higher rate (30.8%) than FPV (26.4%), a higher frequency in younger cats (<3 months) compared to other age classes, and a significant association with the presence of respiratory clinical signs. The observed positivity rate was expected, in line with the widespread diffusion of FCoV, which can reach prevalence rates of up to 90% in the feline population, especially those living in multi-cat environments [46], with kittens being the main fecal shedders [47,48]. While these results were limited to the detection of the FCoV genome, they reflected FCoV excretion by tested cats at introduction into the shelter [13], with potential implications for the spread or evolution of FCoV infection dynamics in this multi-cat environment. However, in this study, viral shedding patterns, including FCoV RNA quantification and duration of shedding, were not assessed after hospitalization, nor was the development of FIP assessed for tested cats. Instead, the association with respiratory clinical signs might be more plausibly linked to the presence and excretion of FCoV even in cats affected by diseases other than coronavirus, stress-related immunosuppression, or confounding by age (young cats are more likely to exhibit both FCoV shedding and respiratory infections due to other causes), rather than to a specific cause–effect relationship.

All detected FCoV strains belonged to type I and, based on the partial fragments of the M and S genes analyzed, they were related to FCoV strains already circulating in the same area [2]. In addition to previous data, these results confirmed the widespread circulation of the FCoV-I type and the almost total absence of specific mutations (M1058L or S1060A) in the spike protein gene. These mutations have been evaluated for diagnostic use in tissue samples and effusions or other fluids, with varying degrees of sensitivity and correlations between cats diagnosed with or without FIP [13]. Instead, baseline data on these mutations in strains from fecal samples are limited: detection and analysis of the M1058L—S1060A mutations in this study were possible for 68.8% (42/61) of FCoV-positive samples. Although a specific diagnosis of FIP was not made in this study, the cats examined here did not show any specific suspicious clinical signs. Therefore, no specific mutation was detected in cats apparently unaffected by FIP. However, specific evaluations need to be performed to confirm these preliminary data.

These specific mutations in the S2 fusion domain of the spike protein gene have been detected in tissues or effusions and have been associated with the development of FIP [13,22] or have been associated with systemic FCoV infection, rather than specifically with FIP [49]. Although RT-PCR for FCoV is performed on fecal samples and is sometimes used to identify cats that shed the FCoV virus, to manage infection in multi-cat households [13], testing to detect these mutations is not commonly performed on this type of sample. Despite being limited to a single test on incoming cats, this study adds novel baseline data regarding FCoV-shedding cats. Furthermore, these results confirm the common presence of non-mutated FCoV in feces [46] but suggest the need to confirm these findings in cats with or without FIP to evaluate this finding according to pathotype, thus prompting further studies.

Some limitations to the study should be acknowledged: (i) this study was limited to a single sampling at the time of introduction of the cats, preventing us from obtaining data on the subsequent clinical evolution of specific diseases and from determining the basic reproduction number (R_0_); while acknowledging the lack of these additional data in this specific case, the shelter serves to ensure adequate care or spaying/neutering, hosting cats only for the time necessary and not for life; (ii) only qualitative PCR/RT-PCR were performed, preventing quantification of viral shedding, and these tests were performed on single samples without monitoring viral shedding after the animal was introduced into the shelter; (iii) the statistical analysis performed was based on an initial clinical examination by official veterinarians, which may have been limited by some difficulties in managing stray cats, by the intent to minimize handling, or by overlapping clinical signs; (iv) the availability of FCoV S gene sequences in public-domain databases, particularly limited for those originating from Italy or other European countries, has prevented a broader comparison, to outline correlations with different origins.

Nevertheless, this study highlights a non-negligible proportion of cats testing positive for *Protoparvovirus carnivoran*1 (26.4%) and feline coronavirus (30.8%) at the time of admission to the shelter facility, emphasizing the need for strict isolation and quarantine measures, as well as for the use of highly sensitive and specific laboratory tests to prevent viral spread within shelters and to allow individual clinical monitoring of positive animals, thereby facilitating the provision of appropriate veterinary care and ensuring animal welfare.

## 5. Conclusions

In conclusion, this study evaluated the detection of FPV/CPV-2 and FCoV in stray or colony cats upon their first arrival at a public shelter. Approximately 30% of cats tested positive for at least one virus, highlighting the need for appropriate biosecurity measures to manage the health of these cats and to prevent the spread of viruses in this multi-cat environment. In some cases, associations with specific risk factors were observed and discussed. These data also provided an analysis of field viral strains circulating in the free-ranging feline population, highlighting both the links between FPV and CPV-2 and the current potential risks for both the domestic cat population and other animal species sharing the same environments. Finally, this study provided baseline data possibly useful for improving the management of cats in shelters and reducing the risk of introducing and spreading potentially serious viral infections.

## Figures and Tables

**Table 1:** Categorization of the associated data considered for this study.

Variable	Category
Origin	Stray Colony
Age	<3 months 3–6 months 6–12 months 12–36 months >36 months
Gender	Males Intact males Spayed males Females Intact females Neutered females
Reason for hospitalization	Pathology Trauma Neutering
Health status	Healthy Gastrointestinal signs Respiratory signs Gastrointestinal and respiratory signs Trauma Miscellaneous

**Table 2:** Amino acid variations in the VP2 sequence of the analyzed FPV/CPV-2c strains.

Virus Type/Variant or Strain	Accession Number	VP2 Amino Acid Residue										
		292	293	297	300	305	309	323	324	370	426	506
^1^ FPV	KX943311	N	S	S	A	D	Q	D	Y	Q	N	L
^1^ CPV-2	M38245	-	-	-	-	-	-	N	-	-	-	-
^1^ CPV-2a	MF177224	-	-	A	G	Y	-	N	-	-	-	-
^1^ CPV-2b	MF177226	-	-	-	G	Y	-	N	-	-	D	-
^1^ CPV-2c	MF177228	-	-	A	G	Y	-	N	-	-	E	-
^1^ CPV-2c (“Asian-like”)	MF510157	-	-	A	G	Y	-	N	I	R	E	-
^2^ FPV_IZSSI_2023PA7283	PZ012444	-	-	-	-	-	-	-	-	-	-	-
^2^ FPV_IZSSI_2023PA7919	PZ012445	-	-	-	-	-	-	-	-	-	-	I
^2^ FPV_IZSSI_2024PA5883	PZ012456	-	-	-	-	-	L	-	-	-	-	-
^2^ FPV_IZSSI_2023PA29335id10	PZ012452	Y	F	-	-	-	-	-	-	-	-	-
^2^ CPV-2c_IZSSI_2024PA12872	PZ012459	-	-	A	G	Y	-	N	I	R	E	-

^1^ Reference sequences; ^2^ Sequences analyzed in this study. Sites showing no variation compared with the first-row sequence are indicated by “-”.

**Table 3:** Estimated FPV, FCoV, or FPV–FCoV coinfection PCR prevalence according to the evaluated variables in the enrolled cat population.

Variable	Parameters	FPV				FCoV				FPV/FCoV Coinfection			
		Nr.	Neg	Pos	% Pos	Nr.	Neg	Pos	% Pos	Nr.	Neg	Pos	% Pos
Origin	Stray	208	148	60	28.8	109	72	37	33.9	109	91	18	16.5
	Colony	160	123	37	23.1	89	65	24	26.9	89	86	3	3.4
Age	< 3 months	10	8	2	20	10	1	9	90	10	8	2	20
	3–6 months	117	98	19	16.2	54	39	15	27.7	54	49	5	9.3
	6–12 months	21	11	10	47.6	13	10	3	23.07	13	12	1	7.7
	12–36 months	151	103	48	31.8	92	64	28	30.4	92	81	11	11.9
	>36 months	69	51	18	26.1	29	23	6	20.7	29	27	2	6.9
Gender	Female	196	154	42	21.4	106	77	29	27.4	106	98	8	7.6
	ntact female	174	138	36	20.7	95	69	26	27.4	95	88	7	7.4
	Neutered female	22	16	6	27.3	11	8	3	27.3	11	10	1	9.1
	Male	172	117	55	32	92	60	32	34.8	92	79	13	14.1
	Intact male	146	98	48	32.9	81	51	30	37	81	68	13	16
	Spayed male	26	19	7	26.9	11	9	2	18.2	11	11	0	0
Reason for hospitalization	Pathology	263	191	72	27.4	131	85	46	35.1	131	114	17	13
	Trauma	76	57	19	25	42	34	8	19	42	39	3	7.1
	Neutering	29	23	6	20.7	25	18	7	28	25	24	1	4
Clinical signs	Healthy	36	27	9	25	33	26	7	21.2	33	32	1	3
	Gastrointestinal	84	60	24	28.6	38	24	14	36.8	38	34	4	10.5
	Respiratory	151	107	44	29.1	68	39	29	42.6	68	54	14	20.6
	Trauma	76	57	19	25	42	34	8	19	42	39	3	7.1
	Miscellaneous	38	29	9	23.7	19	14	5	26.3	19	18	1	5.3

**Table 4:** Univariable analysis of factors associated with FPV positivity, FCoV positivity, and FPV/FCoV coinfection. Bold values indicate statistically significant associations (*p* < 0.05). Odds ratio (OR) and confidence interval (CI) are reported.

Variable	Comparison	FPV OR (95% CI) *p*-Value	FCoV OR (95% CI) *p*-Value	Coinfection OR (95% CI) *p*-Value
Origin	Stray vs. Colony	0.742 (0.462–1.19) 0.234	1.39 (0.754–2.57) 0.354	5.67 (1.61–19.9) **0.002**
Age	<3 vs. 3–6 months	0.776 (0.153–3.94) 0.670	23.4 (2.73–201) **<0.001**	2.45 (0.404–14.9) 0.299
	<3 vs. 6–12 months	3.64 (0.619–21.4) 0.240	30 (2.63–343) **0.003**	3.00 (0.232–38.9) 0.560
	<3 vs. 12–36 months	1.86 (0.381–9.11) 0.725	20.6 (2.49–170) **<0.001**	1.84 (0.346–9.8) 0.612
	<3 vs. >36 months	1.41 (0.274–7.28) 1.000	34.5 (3.63–328) **<0.001**	3.38 (0.408–27.9) 0.267
	3–6 vs. 6–12 months	4.69 (1.75–12.6) **0.003**	1.28 (0.310–5.31) 1.000	1.22 (0.131–11.5) 1.000
	3–6 vs. 12–36 months	2.4 (1.32–4.38) **0.004**	1.28 (0.310–5.31) 1.000	0.751 (0.246–2.29) 0.786
	3–6 vs. >36 months	1.82 (0.879–3.77) 0.129	1.47 (0.502–4.33) 0.600	1.38 (0.250–7.58) 1.000
	6–12 vs. 12–36 months	0.513 (0.204–1.29) 0.217	0.686 (0.175–2.68) 0.751	0.614 (0.0726–5.19) 1.000
	6–12 vs. >36 months	0.388 (0.141–1.07) 0.104	1.15 (0.239–5.54) 1.000	1.13 (0.0928–13.6) 1.000
	12–36 vs. >36 months	1.32 (0.698–2.5) 0.431	0.596 (0.219–1.62) 0.353	0.545 (0.114–2.62) 0.732
Gender	Male vs. Female	0.580 (0.363–0.926) **0.024**	1.42 (0.773–2.59) 0.283	2.02 (0.796–5.11) 0.167
	Intact vs. Neutered female	0.696 (0.254–1.91) 0.581	0.995 (0.245–4.04) 1.000	1.26 (0.140–11.3) 1.000
	Intact vs. Spayed male	1.33 (0.523–3.38) 0.652	0.378 (0.0765–1.87) 0.318	0.221 (0.0122–3.97) 0.353
Reason for hospitalization	Pathology vs. Trauma	0.783 (0.277–2.21) 0.799	0.435 (0.186–1.02) 0.057	0.516 (0.143–1.86) 0.411
	Pathology vs. Neutering	0.692 (0.271–1.77) 0.514	1.39 (0.541–3.58) 0.646	3.58 (0.454–28.2) 0.310
	Trauma vs. Neutering	1.13 (0.630–2.03) 0.769	0.605 (0.189–1.94) 0.546	1.85 (0.182–18.8) 1.000
Clinical signs	Healthy vs. Gastrointestinal	0.833 (0.342–2.03) 0.824	2.17 (0.748–6.28) 0.196	3.76 (0.399–35.5) 0.363
	Healthy vs. Respiratory	0.811 (0.353–1.86) 0.685	2.76 (1.05–7.24) **0.046**	8.30 (1.04–66.1) **0.019**
	Healthy vs. Trauma	1.00 (0.400–2.50) 1.00	0.874 (0.281–2.72) 1.000	2.46 (0.244–24.8) 0.626
	Healthy vs. Miscellaneous	1.07 (0.371–3.11) 1.000	1.33 (0.355–4.96) 0.739	1.78 (0.105–30.2) 1.000
	Respiratory vs. Gastrointestinal	0.854 (0.433–1.68) 0.734	1.38 (0.592–3.24) 0.525	3.78 (0.796–17.9) 0.130

## Data Availability

The nucleotide sequences generated during the current study are available in the GenBank repository under accession numbers PZ012441-PZ012572.

## Supplementary Materials

The following supporting information can be downloaded at: https://www.mdpi.com/article/10.3390/vetsci13060528/s1, Table S1: Sequence of primers, reaction mixes and thermal profiles used for feline panleukopenia/canine parvovirus and feline coronavirus detection; Table S2: Sequence and amplicon size of primers used for feline coronavirus genotyping and partial spike gene amplification, including codons for residues of the S protein affected by mutations M1058L and S1060A, reaction mixes and thermal profiles; Table S3: List of sequences submitted to the DDBJ/EMBL/GenBank databases. Table S4: Nucleotide identities with partial VP2 gene sequences of FPV and CPV-2c strains detected in cats and in a wild carnivore in Italy (at least two sequences with the highest rates of nucleotide identities have been included in this table); Figure S1: Maximum-likelihood tree (General Time Reversible (GTR) model, Gamma distribution (+G) with 5 rate categories, and Invariant (+I) sites; bootstrap 1000 replicates) based on 547 partial S gene sequences of feline coronavirus. Black dots indicate FCoV strains analyzed in this study, while white dots indicate FCoV strains already detected in Italy. Each sequence is indicated with accession number—strain/isolate name—country of origin—year of collection.

## Abbreviations

The following abbreviations are used in this manuscript:

FPV	Feline panleukopenia virus
CPV-2	Canine parvovirus type 2
FCoV	Feline coronavirus
PCR	Polymerase chain reaction
