# Supplementary material for: Prevalence of Protoparvovirus carnivoran1/Feline Coronavirus and Associated Risk Factors in Cats Admitted to a Public Shelter in Southern Italy
Source: Vet Sci. 2026 May 29;13(6):528. doi: 10.3390/vetsci13060528 (PMC13307580; doi:10.3390/vetsci13060528)
Supplement: Supplementary file 1 [file vetsci-13-00528-s001.zip › Supplementary Material - Table S1.pdf]

Supplementary Material - Table S1. Sequence of primers, reaction mixes and thermal profiles used for feline panleukopenia/canine parvovirus and feline coronavirus detection.

Virus	PCR assay	Amplification kit	Primer/probe name	Primer/probe sequence (5'-3')	Reference	Reaction mix	Thermal profile
FPV/CPV-2	PCR	GoTaq® G2 DNA Polymerase (Promega)	VP2-850-Forward<br>VP2-1550-Reverse	GAGCATTGGGCTTACCA<br>GCAAGATGCATCAGGATC	[18]	10 µL of 5X Colorless GoTaq® Reaction Buffer<br>1 µL of MgCl2 (25 mM)<br>1 µL of dNTP mix (10 mM)<br>0.5 µL of VP2-850-Forward primer (50 µM)<br>0.5 µL of VP2-1550-Reverse primer (50 µM)<br>0.25 µL of GoTaq® G2 DNA Polymerase (5u/µl)<br>5 µL of DNA extract<br>31.75 µL of nuclease-free water	1 cycle at 94 °C for 2 min<br>40 cycles at 94 °C for 30 s<br>55 °C for 60 s<br>72 °C for 60 s<br>1 cycle at 72 °C for 10 min
FCoV	RT-PCR	OneStep RT-PCR Kit (Qiagen)	CCV1<br>CCV2	TCCAGATATGTAATGTTCGG<br>TCTGTTGAGTAATCACCAGCT	[20]	10 µL of 5x QIAGEN OneStep RT-PCR Buffer<br>1 µL of CCV1 primer (20 µM)<br>1 µL of CCV2 primer (20 µM),<br>2 µL of (10 mM) dNTP Mix<br>0.22 µL of RNase Inhibitor (40 U/µL)[1]<br>2.5 µL of RNA extract<br>31.28 µL of nuclease-free water	1 cycle at 94 °C for 2 min<br>40 cycles at 94 °C for 30 s<br>55 °C for 60 s<br>72 °C for 60 s<br>1 cycle at 72 °C for 10 min

[1]Provided by Euroclone S.p.A., Pero, Italy.
