# Supplementary material for: Prevalence of Protoparvovirus carnivoran1/Feline Coronavirus and Associated Risk Factors in Cats Admitted to a Public Shelter in Southern Italy
Source: Vet Sci. 2026 May 29;13(6):528. doi: 10.3390/vetsci13060528 (PMC13307580; doi:10.3390/vetsci13060528)
Supplement: Supplementary file 1 [file vetsci-13-00528-s001.zip › Supplementary Material - Table S2.pdf]

Supplementary Material – Table S2. Sequence and amplicon size of primers used for feline coronavirus genotyping and partial spike gene amplification, including codons for residues of the S protein affected by mutations M1058L and S1060A, reaction mixes and thermal profiles.

Assay	Step	Amplification kit	Primer	Sequence (5′ to 3′)	Amplicon size	Ref.	Reaction mix	Thermal profile
FCoV genotyping	RT-PCR	SuperScript™ One-Step RT-PCR System with Platinum™ Taq DNA Polymerase (ThermoFisher Scientific, Monza, Italy)	Iffs	GTTTCAACCTAGAAAGCCTCAGAT	376 (FCoV-I)<br>282 (FCoV-II)	[21]	12.5 μL of 2X Reaction Mix<br>1 μL of Iffs primer (20 μM)<br>1 μL of Icfs primer (20 μM)<br>1 μL of Iubs primer (20 μM)<br>0.5 μL of RT/Platinum™ Taq Mix<br>2 μL of RNA extract<br>7 μL of nuclease-free water	1 cycle at 42 °C for 60 min<br>1 cycle at 94 °C for 2 min<br>35 cycles at 94 °C for 60 s<br>50 °C for 60 s<br>68 °C for 60 s<br>1 cycle at 68 °C for 10 min
			Icfs	GCCTAGTATTATACCTGACTA				
			Iubs	CCACACATACCAAGGCC				
	Nested PCR	GoTaq® G2 DNA Polymerase Kit (Promega Corporation, Madison, WI, USA)	nIffles	CCTAGAAAGCCTCAGATGAGTG	360 (FCoV-I)<br>211 (FCoV-II)		5 μL of 5X Colorless GoTaq® Reaction Buffer<br>0.5 μL of dNTP mix (10 mM)<br>1 μL of nIffles primer (20 μM)<br>1 μL of nIcfs primer (20 μM)<br>1 μL of nIubs primer (20 μM)<br>0.25 μL of GoTaq® G2 DNA Polymerase<br>1 μL of cDNA<br>15.25 μL of nuclease-free water	1 cycle at 94 °C for 2 min<br>35 cycles at 94 °C for 30 s<br>47 °C for 60 s<br>72 °C for 60 s<br>1 cycle at 72 °C for 10 min
			nIcfs	CAGACCAAACTGGACTGTAC				
			nIubs	CCAAGGCCATTTTACATA				
Partial FCoV S gene amplification	RT-PCR	SuperScript™ One-Step RT-PCR System with Platinum™ Taq DNA Polymerase	UCD5F	GCCCAATATTACAATGGCATAA	215 (FCoV-I)	[22]	12.5 μL of 2X Reaction Mix<br>0.5 μL of UCD5F primer (50 μM)<br>0.5 μL of UCD3248R primer (50 μM)<br>or<br>0.5 μL of G2F primer (50 μM)<br>0.5 μL of G2R primer (50 μM)<br>0.5 μL of RT/Platinum™ Taq Mix<br>2 μL of RNA extract<br>9 μL of nuclease-free water	1 cycle at 50 °C for 30 min<br>1 cycle at 94 °C for 2 min<br>45 cycles at 94 °C for 30 s<br>50 °C for 30 s<br>68 °C for 45 s<br>1 cycle at 72 °C for 10 min
			UCD3248R	AAGGCATTAGCAAGTATTTTC				
	RT-PCR		G2F	TAGGTGCACTTGGTGGTGGT	250 (FCoV-II)			
			G2R	GCATTTGCAAGTGAAAACAAA				
