# Supplementary material for: Prevalence of Protoparvovirus carnivoran1/Feline Coronavirus and Associated Risk Factors in Cats Admitted to a Public Shelter in Southern Italy
Source: Vet Sci. 2026 May 29;13(6):528. doi: 10.3390/vetsci13060528 (PMC13307580; doi:10.3390/vetsci13060528)
Supplement: Supplementary file 1 [file vetsci-13-00528-s001.zip › Supplementary Material - Table S3.pdf]

Supplementary Material – Table S3. List of sequences submitted to the DDBJ/EMBL/GenBank databases.

Sequence_ID	Collection_date	Genotype	Gene	Accession nr.
FPV_IZSSI_2023PA5596	06-Mar-2023	FPV	VP2	PZ012441
FPV_IZSSI_2023PA6186	10-Mar-2023	FPV	VP2	PZ012442
FPV_IZSSI_2023PA6640	15-Mar-2023	FPV	VP2	PZ012443
FPV_IZSSI_2023PA7283	20-Mar-2023	FPV	VP2	PZ012444
FPV_IZSSI_2023PA7919	23-Mar-2023	FPV	VP2	PZ012445
FPV_IZSSI_2023PA10441	11-Apr-2023	FPV	VP2	PZ012446
FPV_IZSSI_2023PA10928	17-Apr-2023	FPV	VP2	PZ012447
FPV_IZSSI_2023PA15754	31-May-2023	FPV	VP2	PZ012448
FPV_IZSSI_2023PA17703	20-Jun-2023	FPV	VP2	PZ012449
FPV_IZSSI_2023PA22584	16-Aug-2023	FPV	VP2	PZ012450
FPV_IZSSI_2023PA25781	20-Sep-2023	FPV	VP2	PZ012451
FPV_IZSSI_2023PA29335id10	26-Oct-2023	FPV	VP2	PZ012452
FPV_IZSSI_2023PA29335id33	26-Oct-2023	FPV	VP2	PZ012453
FPV_IZSSI_2024PA4763	22-Feb-2024	FPV	VP2	PZ012454
FPV_IZSSI_2024PA5048	27-Feb-2024	FPV	VP2	PZ012455
FPV_IZSSI_2024PA5883	03-Mar-2024	FPV	VP2	PZ012456
FPV_IZSSI_2024PA5985	06-Mar-2024	FPV	VP2	PZ012457
FPV_IZSSI_2024PA29858	22-Oct-2024	FPV	VP2	PZ012458
CPV-2c_IZSSI_2024PA12872	09-May-2024	CPV-2c	VP2	PZ012459
CPV-2c_IZSSI_2024PA15872	30-May-2024	CPV-2c	VP2	PZ012460
FCoV_M_gene_IZSSI_2023PA5587	06-Mar-2023	FCoV	M	PZ012461
FCoV_M_gene_IZSSI_2023PA5588	06-Mar-2023	FCoV	M	PZ012462
FCoV_M_gene_IZSSI_2023PA5993	09-Mar-2023	FCoV	M	PZ012463
FCoV_M_gene_IZSSI_2023PA6186	10-Mar-2023	FCoV	M	PZ012464
FCoV_M_gene_IZSSI_2023PA6639	15-Mar-2023	FCoV	M	PZ012465
FCoV_M_gene_IZSSI_2023PA7919	23-Mar-2023	FCoV	M	PZ012466
FCoV_M_gene_IZSSI_2023PA7922	23-Mar-2023	FCoV	M	PZ012467
FCoV_M_gene_IZSSI_2023PA8704	29-Mar-2023	FCoV	M	PZ012468
FCoV_M_gene_IZSSI_2023PA10445	11-Apr-2023	FCoV	M	PZ012469
FCoV_M_gene_IZSSI_2023PA10724	14-Apr-2023	FCoV	M	PZ012470
FCoV_M_gene_IZSSI_2023PA10928	17-Apr-2023	FCoV	M	PZ012471
FCoV_M_gene_IZSSI_2023PA10929	17-Apr-2023	FCoV	M	PZ012472
FCoV_M_gene_IZSSI_2023PA14295	17-May-2023	FCoV	M	PZ012473
FCoV_M_gene_IZSSI_2023PA14380	17-May-2023	FCoV	M	PZ012474
FCoV_M_gene_IZSSI_2023PA14381	17-May-2023	FCoV	M	PZ012475
FCoV_M_gene_IZSSI_2023PA14765	23-May-2023	FCoV	M	PZ012476
FCoV_M_gene_IZSSI_2023PA15164	24-May-2023	FCoV	M	PZ012477
FCoV_M_gene_IZSSI_2023PA15744	31-May-2023	FCoV	M	PZ012478
FCoV_M_gene_IZSSI_2023PA15747	31-May-2023	FCoV	M	PZ012479
FCoV_M_gene_IZSSI_2023PA15748	31-May-2023	FCoV	M	PZ012480
FCoV_M_gene_IZSSI_2023PA15752	31-May-2023	FCoV	M	PZ012481
FCoV_M_gene_IZSSI_2023PA15756	31-May-2023	FCoV	M	PZ012482
FCoV_M_gene_IZSSI_2023PA15758	31-May-2023	FCoV	M	PZ012483
FCoV_M_gene_IZSSI_2023PA15906	31-May-2023	FCoV	M	PZ012484
FCoV_M_gene_IZSSI_2023PA15907	31-May-2023	FCoV	M	PZ012485
FCoV_M_gene_IZSSI_2023PA16692	09-Jun-2023	FCoV	M	PZ012486
FCoV_M_gene_IZSSI_2023PA17022	13-Jun-2023	FCoV	M	PZ012487
FCoV_M_gene_IZSSI_2023PA17024	13-Jun-2023	FCoV	M	PZ012488
FCoV_M_gene_IZSSI_2023PA17568	16-Jun-2023	FCoV	M	PZ012489

FCoV_M_gene_IZSSI_2023PA18110	23-Jun-2023	FCoV	M	PZ012490
FCoV_M_gene_IZSSI_2023PA18112	23-Jun-2023	FCoV	M	PZ012491
FCoV_M_gene_IZSSI_2023PA18898	30-Jun-2023	FCoV	M	PZ012492
FCoV_M_gene_IZSSI_2024PA8177	27-Mar-2024	FCoV	M	PZ012493
FCoV_M_gene_IZSSI_2024PA8183	27-Mar-2024	FCoV	M	PZ012494
FCoV_M_gene_IZSSI_2024PA12872	09-May-2024	FCoV	M	PZ012495
FCoV_M_gene_IZSSI_2024PA14155	20-May-2024	FCoV	M	PZ012496
FCoV_M_gene_IZSSI_2024PA14157	20-May-2024	FCoV	M	PZ012497
FCoV_M_gene_IZSSI_2024PA15870	30-May-2024	FCoV	M	PZ012498
FCoV_M_gene_IZSSI_2024PA15880	30-May-2024	FCoV	M	PZ012499
FCoV_M_gene_IZSSI_2024PA18710	01-Jul-2024	FCoV	M	PZ012500
FCoV_M_gene_IZSSI_2024PA19259	05-Jul-2024	FCoV	M	PZ012501
FCoV_M_gene_IZSSI_2024PA28891	18-Oct-2024	FCoV	M	PZ012502
FCoV_M_gene_IZSSI_2024PA28897	18-Oct-2024	FCoV	M	PZ012503
FCoV_M_gene_IZSSI_2024PA32114	15-Nov-2024	FCoV	M	PZ012504
FCoV_M_gene_IZSSI_2024PA34311	16-Dec-2024	FCoV	M	PZ012505
FCoV-I_S_gene_IZSSI_2023PA5587	06-Mar-2023	FCoV-I	S	PZ012506
FCoV-I_S_gene_IZSSI_2023PA5588	06-Mar-2023	FCoV-I	S	PZ012507
FCoV-I_S_gene_IZSSI_2023PA5601	06-Mar-2023	FCoV-I	S	PZ012508
FCoV-I_S_gene_IZSSI_2023PA5993	09-Mar-2023	FCoV-I	S	PZ012509
FCoV-I_S_gene_IZSSI_2023PA6186	10-Mar-2023	FCoV-I	S	PZ012510
FCoV-I_S_gene_IZSSI_2023PA6639	15-Mar-2023	FCoV-I	S	PZ012511
FCoV-I_S_gene_IZSSI_2023PA6640	15-Mar-2023	FCoV-I	S	PZ012512
FCoV-I_S_gene_IZSSI_2023PA7922	23-Mar-2023	FCoV-I	S	PZ012513
FCoV-I_S_gene_IZSSI_2023PA8704	29-Mar-2023	FCoV-I	S	PZ012514
FCoV-I_S_gene_IZSSI_2023PA10445	11-Apr-2023	FCoV-I	S	PZ012515
FCoV-I_S_gene_IZSSI_2023PA10724	14-Apr-2023	FCoV-I	S	PZ012516
FCoV-I_S_gene_IZSSI_2023PA10928	17-Apr-2023	FCoV-I	S	PZ012517
FCoV-I_S_gene_IZSSI_2023PA10929	17-Apr-2023	FCoV-I	S	PZ012518
FCoV-I_S_gene_IZSSI_2023PA10930	17-Apr-2023	FCoV-I	S	PZ012519
FCoV-I_S_gene_IZSSI_2023PA14295	17-May-2023	FCoV-I	S	PZ012520
FCoV-I_S_gene_IZSSI_2023PA14381	17-May-2023	FCoV-I	S	PZ012521
FCoV-I_S_gene_IZSSI_2023PA14765	23-May-2023	FCoV-I	S	PZ012522
FCoV-I_S_gene_IZSSI_2023PA15164	24-May-2023	FCoV-I	S	PZ012523
FCoV-I_S_gene_IZSSI_2023PA15744	31-May-2023	FCoV-I	S	PZ012524
FCoV-I_S_gene_IZSSI_2023PA15750	31-May-2023	FCoV-I	S	PZ012525
FCoV-I_S_gene_IZSSI_2023PA15752	31-May-2023	FCoV-I	S	PZ012526
FCoV-I_S_gene_IZSSI_2023PA15756	31-May-2023	FCoV-I	S	PZ012527
FCoV-I_S_gene_IZSSI_2023PA15906	31-May-2023	FCoV-I	S	PZ012528
FCoV-I_S_gene_IZSSI_2023PA15907	31-May-2023	FCoV-I	S	PZ012529
FCoV-I_S_gene_IZSSI_2023PA16478	07-Jun-2023	FCoV-I	S	PZ012530
FCoV-I_S_gene_IZSSI_2023PA17022	13-Jun-2023	FCoV-I	S	PZ012531
FCoV-I_S_gene_IZSSI_2023PA17024	13-Jun-2023	FCoV-I	S	PZ012532
FCoV-I_S_gene_IZSSI_2023PA17568	16-Jun-2023	FCoV-I	S	PZ012533
FCoV-I_S_gene_IZSSI_2023PA18110	23-Jun-2023	FCoV-I	S	PZ012534
FCoV-I_S_gene_IZSSI_2023PA18115	23-Jun-2023	FCoV-I	S	PZ012535
FCoV-I_S_gene_IZSSI_2023PA18898	30-Jun-2023	FCoV-I	S	PZ012536
FCoV-I_S_gene_IZSSI_2024PA8177	27-Mar-2024	FCoV-I	S	PZ012537
FCoV-I_S_gene_IZSSI_2024PA11146	22-Apr-2024	FCoV-I	S	PZ012538
FCoV-I_S_gene_IZSSI_2024PA12872	09-May-2024	FCoV-I	S	PZ012539
FCoV-I_S_gene_IZSSI_2024PA14157	20-May-2024	FCoV-I	S	PZ012540
FCoV-I_S_gene_IZSSI_2024PA15880	30-May-2024	FCoV-I	S	PZ012541

FCoV-I_S_gene_IZSSI_2024PA18710	01-Jul-2024	FCoV-I	S	PZ012542
FCoV-I_S_gene_IZSSI_2024PA19259	05-Jul-2024	FCoV-I	S	PZ012543
FCoV-I_S_gene_IZSSI_2024PA28891	18-Oct-2024	FCoV-I	S	PZ012544
FCoV-I_S_gene_IZSSI_2024PA28897	18-Oct-2024	FCoV-I	S	PZ012545
FCoV-I_S_gene_IZSSI_2024PA28909	18-Oct-2024	FCoV-I	S	PZ012546
FCoV-I_S_gene_IZSSI_2024PA32114	15-Nov-2024	FCoV-I	S	PZ012547
FCoV-I_S_gene_IZSSI_2024PA34311	16-Dec-2024	FCoV-I	S	PZ012548
FCoV_S_gene_IZSSI_2023PA5587	06-Mar-2023	FCoV	S	PZ012549
FCoV_S_gene_IZSSI_2023PA5588	06-Mar-2023	FCoV	S	PZ012550
FCoV_S_gene_IZSSI_2023PA5601	06-Mar-2023	FCoV	S	PZ012551
FCoV_S_gene_IZSSI_2023PA5993	09-Mar-2023	FCoV	S	PZ012552
FCoV_S_gene_IZSSI_2023PA6186	10-Mar-2023	FCoV	S	PZ012553
FCoV_S_gene_IZSSI_2023PA6639	15-Mar-2023	FCoV	S	PZ012554
FCoV_S_gene_IZSSI_2023PA7919	23-Mar-2023	FCoV	S	PZ012555
FCoV_S_gene_IZSSI_2023PA8704	29-Mar-2023	FCoV	S	PZ012556
FCoV_S_gene_IZSSI_2023PA10928	17-Apr-2023	FCoV	S	PZ012557
FCoV_S_gene_IZSSI_2023PA10929	17-Apr-2023	FCoV	S	PZ012558
FCoV_S_gene_IZSSI_2023PA10930	17-Apr-2023	FCoV	S	PZ012559
FCoV_S_gene_IZSSI_2023PA14295	17-May-2023	FCoV	S	PZ012560
FCoV_S_gene_IZSSI_2023PA14381	17-May-2023	FCoV	S	PZ012561
FCoV_S_gene_IZSSI_2023PA14765	23-May-2023	FCoV	S	PZ012562
FCoV_S_gene_IZSSI_2023PA15744	31-May-2023	FCoV	S	PZ012563
FCoV_S_gene_IZSSI_2023PA15752	31-May-2023	FCoV	S	PZ012564
FCoV_S_gene_IZSSI_2023PA15756	31-May-2023	FCoV	S	PZ012565
FCoV_S_gene_IZSSI_2023PA17022	13-Jun-2023	FCoV	S	PZ012566
FCoV_S_gene_IZSSI_2023PA18110	23-Jun-2023	FCoV	S	PZ012567
FCoV_S_gene_IZSSI_2023PA18112	23-Jun-2023	FCoV	S	PZ012568
FCoV_S_gene_IZSSI_2024PA14157	20-May-2024	FCoV	S	PZ012569
FCoV_S_gene_IZSSI_2024PA15880	30-May-2024	FCoV	S	PZ012570
FCoV_S_gene_IZSSI_2024PA19259	05-Jul-2024	FCoV	S	PZ012571
FCoV_S_gene_IZSSI_2024PA28891	18-Oct-2024	FCoV	S	PZ012572
