# Supplementary material for: Prevalence of Protoparvovirus carnivoran1/Feline Coronavirus and Associated Risk Factors in Cats Admitted to a Public Shelter in Southern Italy
Source: Vet Sci. 2026 May 29;13(6):528. doi: 10.3390/vetsci13060528 (PMC13307580; doi:10.3390/vetsci13060528)
Supplement: Supplementary file 1 [file vetsci-13-00528-s001.zip › Supplementary Material - Table S4.pdf]

Supplementary Material – Table S4. Nucleotide identities of partial VP2 gene sequences of FPV and CPV-2c strains obtained in this study in relation to previously published sequences originating from viruses detected in domestic and wild carnivore in Italy (at least two sequences with the highest rates of nucleotide identities have been included in this table).

Strain	Acc.nr.	Nucleotide identity	Acc.nr.	Isolate/strain name	Host	Country	Year
				**FPV**			
FPV_IZSSI_2023PA7283	PZ012444	99.86%	PP351396	PPVC1/IZSSI_2021PA59131	Cat	Italy (Sicily)	2021
		99.43%	MW847180	37039	Cat	Italy (Veneto)	2017
			MW847158	398781	Cat	Italy (Veneto)	2011
			OP588006	IZSVe_21/29010-6_golden_jackal_ITA	Golden jackal	Italy (FVG*)	2021
FPV_IZSSI_2023PA7919	PZ012445	99.71%	PP351396	PPVC1/IZSSI_2021PA59131	Cat	Italy (Sicily)	2021
		99.29%	OP588006	IZSVe_21/29010-6_golden_jackal_ITA	Golden jackal	Italy (FVG*)	2021
			MW847174	36891	Cat	Italy (Veneto)	2016
			MW847181	37038	Cat	Italy (Veneto)	2017
FPV_IZSSI_2023PA17703	PZ012449	99.57%	OP588006	IZSVe_21/29010-6_golden_jackal_ITA	Golden jackal	Italy (FVG*)	2021
			MW847174	36891	Cat	Italy (Veneto)	2016
			MW847181	37038	Cat	Italy (Veneto)	2017
FPV_IZSSI_2024PA4763	PZ012454	99.71%	KX434461	FPV_IZSSI_3201_1_15	Cat	Italy (Sicily)	2015
			MW847174	36891	Cat	Italy (Veneto)	2016
			MW847187	880007	Cat	Italy (Veneto)	2017
			MW847181	37038	Cat	Italy (Veneto)	2017
			MW847158	398781	Cat	Italy (Veneto)	2011
FPV_IZSSI_2023PA5596	PZ012441	100%	PP351396	PPVC1/IZSSI_2021PA59131	Cat	Italy (Sicily)	2021
		99.86%	PP351432	PPVC1/IZSSI_2022PA27325	Cat	Italy (Sicily)	2022
FPV_IZSSI_2023PA6640	PZ012443	99.86%	PP351396	PPVC1/IZSSI_2021PA59131	Cat	Italy (Sicily)	2021
		99.71%	PP351432	PPVC1/IZSSI_2022PA27325	Cat	Italy (Sicily)	2022
FPV_IZSSI_2023PA10441	PZ012446	99.86%	PP351396	PPVC1/IZSSI_2021PA59131	Cat	Italy (Sicily)	2021
		99.71%	PP351432	PPVC1/IZSSI_2022PA27325	Cat	Italy (Sicily)	2022
FPV_IZSSI_2023PA10928	PZ012447	99.86%	PP351396	PPVC1/IZSSI_2021PA59131	Cat	Italy (Sicily)	2021
		99.71%	PP351432	PPVC1/IZSSI_2022PA27325	Cat	Italy (Sicily)	2022
FPV_IZSSI_2023PA15754	PZ012448	99.71%	MW847115	380915	Cat	Italy (Veneto)	2011
			PP351401	PPVC1/IZSSI_2021PA61198	Cat	Italy (Sicily)	2021
			PP351396	PPVC1/IZSSI_2021PA59131	Cat	Italy (Sicily)	2021
FPV_IZSSI_2023PA25781	PZ012451	100%	PP351401	PPVC1/IZSSI_2021PA61198	Cat	Italy (Sicily)	2021

		99.86%	MW847174	36891	Cat	Italy (Veneto)	2016
			MW847180	37039	Cat	Italy (Veneto)	2017
			MW847187	880007	Cat	Italy (Veneto)	2017
			MW847181	37038	Cat	Italy (Veneto)	2017
			KX434461	FPV_IZSSI_3201_1_15	Cat	Italy (Sicily)	2015
FPV_IZSSI_2024PA29858	PZ012458	99.86%	PP351396	PPVC1/IZSSI_2021PA59131	Cat	Italy (Sicily)	2021
		99.71%	PP351432	PPVC1/IZSSI_2022PA27325	Cat	Italy (Sicily)	2022
FPV_IZSSI_2024PA5883	PZ012456	99.57%	PP351401	PPVC1/IZSSI_2021PA61198	Cat	Italy (Sicily)	2021
			PP351396	PPVC1/IZSSI_2021PA59131	Cat	Italy (Sicily)	2021
		99.43%	MW847174	36891	Cat	Italy (Veneto)	2016
			MW847180	37039	Cat	Italy (Veneto)	2017
			MW847187	880007	Cat	Italy (Veneto)	2017
			MW847181	37038	Cat	Italy (Veneto)	2017
			MW847158	398781	Cat	Italy (Veneto)	2011
			KX434461	FPV_IZSSI_3201_1_15	Cat	Italy (Sicily)	2015
FPV_IZSSI_2023PA22584	PZ012450	99.71%	MW847115	380915	Cat	Italy (Veneto)	2011
		99.57%	MW847199	44350	Cat	Italy (Veneto)	2018
			MW847174	36891	Cat	Italy (Veneto)	2016
			MW847180	37039	Cat	Italy (Veneto)	2017
			MW847187	880007	Cat	Italy (Veneto)	2017
			MW847181	37038	Cat	Italy (Veneto)	2017
			MW847158	398781	Cat	Italy (Veneto)	2011
			KX434461	FPV_IZSSI_3201_1_15	Cat	Italy (Sicily)	2015
FPV_IZSSI_2024PA5048	PZ012455	99.86%	MW847174	36891	Cat	Italy (Veneto)	2016
			MW847180	37039	Cat	Italy (Veneto)	2017
			MW847187	880007	Cat	Italy (Veneto)	2017
			MW847181	37038	Cat	Italy (Veneto)	2017
			MW847158	398781	Cat	Italy (Veneto)	2011
			KX434461	FPV_IZSSI_3201_1_15	Cat	Italy (Sicily)	2015
FPV_IZSSI_2023PA29335id10	PZ012452	99.43%	MW847174	36891	Cat	Italy (Veneto)	2016
			MW847180	37039	Cat	Italy (Veneto)	2017
			MW847187	880007	Cat	Italy (Veneto)	2017
			MW847181	37038	Cat	Italy (Veneto)	2017
			MW847158	398781	Cat	Italy (Veneto)	2011
			KX434461	FPV_IZSSI_3201_1_15	Cat	Italy (Sicily)	2015

FPV_IZSSI_2024PA5985	PZ012457	99.71%	MW847174	36891	Cat	Italy (Veneto)	2016
			MW847180	37039	Cat	Italy (Veneto)	2017
			MW847187	880007	Cat	Italy (Veneto)	2017
			MW847181	37038	Cat	Italy (Veneto)	2017
			MW847158	398781	Cat	Italy (Veneto)	2011
			KX434461	FPV_IZSSI_3201_1_15	Cat	Italy (Sicily)	2015
FPV_IZSSI_2023PA6186	PZ01244	99.86%	MW847174	36891	Cat	Italy (Veneto)	2016
			MW847180	37039	Cat	Italy (Veneto)	2017
			MW847187	880007	Cat	Italy (Veneto)	2017
			MW847181	37038	Cat	Italy (Veneto)	2017
			MW847158	398781	Cat	Italy (Veneto)	2011
			KX434461	FPV_IZSSI_3201_1_15	Cat	Italy (Sicily)	2015
FPV_IZSSI_2023PA29335id33	PZ012453	99.71%	MW847174	36891	Cat	Italy (Veneto)	2016
			MW847180	37039	Cat	Italy (Veneto)	2017
			MW847187	880007	Cat	Italy (Veneto)	2017
			MW847181	37038	Cat	Italy (Veneto)	2017
			MW847158	398781	Cat	Italy (Veneto)	2011
			KX434461	FPV_IZSSI_3201_1_15	Cat	Italy (Sicily)	2015
				**CPV-2c**			
CPV-2c_IZSSI_2024PA12872	PZ012459	100%	OP588002	IZSVe_21/31130-1_dog_ITA	Dog	Italy (FVG*)	2021
CPV-2c_IZSSI_2024PA15872	PZ012460		OR463584	IZSSI_2022PA29683	Dog	Italy (Sicily)	2022
			OR463658	IZSSI_2020PA53415	Dog	Italy (Sicily)	2020
			OR463670	IZSSI_2020PA91431IdMin	Dog	Italy (Sicily)	2020

*Friuli-Venezia Giulia
