# Supplementary material for: Prevalence of Protoparvovirus carnivoran1/Feline Coronavirus and Associated Risk Factors in Cats Admitted to a Public Shelter in Southern Italy
Source: Vet Sci. 2026 May 29;13(6):528. doi: 10.3390/vetsci13060528 (PMC13307580; doi:10.3390/vetsci13060528)

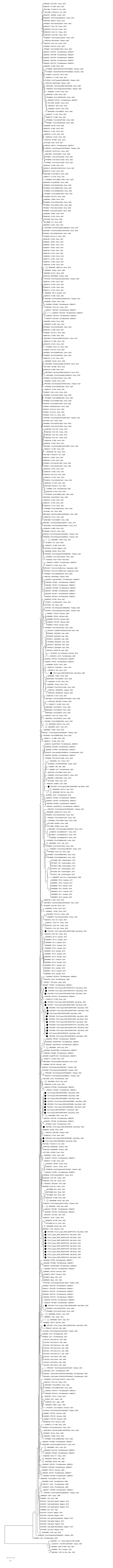

Supplement: Supplementary file 1 [file Supplementary Material - Figure S1.pdf › Supplementary Material - Figure S1.pdf]
